# Attitudes of cancer patients toward ctDNA‐based liquid biopsy for follow‐up

**DOI:** 10.1002/1878-0261.70352

**Published:** 2026-10-21

**Authors:** Siddharth Chhajlani, Laura Mariën, Caro De Weerdt, Sanne Wouters, Laure‐Anne Teuwen, Hans Prenen, Katleen Janssens, Timon Vandamme

**Affiliations:** ^1^ Department of Oncology Antwerp University Hospital Edegem Belgium; ^2^ Center of Medical Genetics University of Antwerp Edegem Belgium; ^3^ Center for Oncological Research University of Antwerp Wilrijk Belgium; ^4^ Department of Medical Genetics Antwerp University Hospital Edegem Belgium

**Keywords:** circulating tumor DNA, liquid biopsy, patient perspectives, shared decision‐making

## Abstract

Circulating tumor DNA‐based liquid biopsy is increasingly integrated into cancer care, particularly for detecting minimal residual disease, yet patient perspectives remain poorly understood. This single‐center, cross‐sectional, mixed‐methods study assessed recall, knowledge, and attitudes toward liquid biopsy and circulating tumor DNA among 195 patients (median age 65 years; 63% female) undergoing cancer treatment or follow‐up, supplemented by focus groups. Although 65% reported prior liquid biopsy, this was confirmed in only 44%, and only 10% had heard of circulating tumor DNA (ctDNA). Correct recall and knowledge on ctDNA were higher among patients with neuroendocrine neoplasms and those with postgraduate education. In hypothetical scenarios assuming proven clinical validity and utility, 62–80% supported liquid biopsy‐guided clinical action, 74% were comfortable with liquid biopsy replacing tissue biopsy, and 79% accepted its use to guide adjuvant treatment. Most patients anticipated reassurance rather than stress, and 85% expected improved quality of life with home‐ or general practitioner‐based blood sampling. Overall, patients showed high acceptance of clinically actionable liquid biopsy use, driven by trust and perceived clinical benefit.

## Graphical abstract

This mixed‐methods study explored cancer patients' experiences, knowledge, and preferences regarding ctDNA‐based liquid biopsy for follow‐up. Awareness of ctDNA was low and recall of previous liquid biopsy was imperfect, yet acceptance was high. Patients valued less invasive sampling, treatment guidance, access to results, and convenient blood collection, emphasizing the need for reliable testing and clear, patient‐centered information. Figure made with Adobe Illustrator.

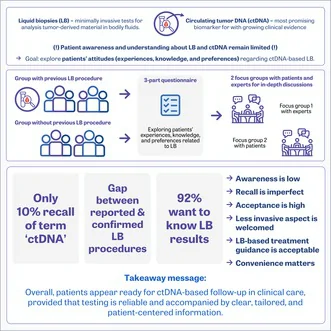

**Abbreviations**
aOR: adjusted odds ratio
ASCO: American Society of Clinical Oncology
CI: confidence interval
ctDNA: circulating tumor DNA
ESMO: European Society for Medical Oncology
GDPR: General Data Protection Regulation
GI: gastrointestinal
GYN: gynecological
HCP: health care professional
LB: liquid biopsy
MRD: minimal residual disease
NEN: neuroendocrine neoplasm
OR: odds ratio
PD: progressive disease
PRRT: peptide receptor radionuclide therapy
QoL: quality of life
SSA: somatostatin analog
TB: tissue biopsy
UZA: Antwerp University Hospital

## 1 Introduction

Liquid biopsies (LBs) are gaining increasing attention as a promising approach for advancing personalized cancer care. LBs, defined as the sampling and molecular analysis of tumor‐derived material in body fluids, offer a minimally invasive means of capturing tumor biology in real time [1]. Blood is the preferred source given its convenience and broad range of circulating tumor‐derived analytes [2]. Established tumor markers like prostate‐specific antigen and carcino‐embryonic antigen remain widely used, despite their suboptimal accuracy [3]. More recently, increasing attention has focused on tumor‐derived cell‐free nucleic acids, particularly circulating tumor DNA (ctDNA), which is released into the bloodstream and carries tumor‐specific molecular alterations [4, 5].

By reflecting the tumor's genomic and epigenomic landscape, ctDNA offers a powerful tool for monitoring tumor burden [6, 7], detecting minimal residual disease (MRD) [8, 9, 10, 11] and identifying therapy resistance [12, 13]. Given these expanding applications [14, 15, 16], ctDNA‐based LBs are increasingly being explored as a complement to tissue‐ and imaging‐based strategies, as also reflected in recent ESMO guidelines [17] and the ASCO clinical guideline draft [18]. Preferences among health care professionals (HCP) align with this shift, as recent evidence indicates a preference for LBs over tissue, reflecting their growing relevance [19].

Existing research has focused mainly on assay performance, clinician readiness, and regulatory considerations, with limited attention to patient experience, knowledge, attitudes, and preferences. A survey by Cancer Patients Europe in over 1200 breast cancer patients revealed a substantial lack of knowledge of LBs, with 60% unaware that LBs could provide reliable disease information [20]. This aligns with prior evidence reporting limited understanding of cancer‐specific blood‐based biomarkers among patients [21]. Yet, trade‐off scenario testing revealed that 90% of patients preferred LBs over tissue biopsy at baseline [21], with test accuracy and turnaround time driving preferences. These findings suggest that assay‐related determinants indirectly shape patients' perceptions, but data addressing these remain scarce. This gap is notable, as successful clinical implementation depends on patient acceptance, alongside technical validity and expert endorsement. Incorporating the patient perspective is essential to ensure that technological advances translate into meaningful improvements in practice. Understanding patients' expectations, knowledge gaps, and perspectives is key to fostering trust, adherence, and ultimately effective implementation [22].

This study was designed to address this gap by surveying a large cohort of cancer patients to investigate patients' recall, experience, knowledge, attitude, and preferences regarding ctDNA‐based LBs.

## 2 Materials and methods

### 2.1 Study design

This single center, cross‐sectional, mixed‐methods study, conducted at the Department of Oncology of the Antwerp University Hospital (UZA, Belgium), combined questionnaire and qualitative focus groups to investigate patients' perspectives on LBs among Dutch‐speaking patients under active oncological follow‐up or treatment. The objectives were to explore patients' recall, experiences, knowledge, attitude, and preferences related to ctDNA‐based LB. Adult patients aged ≥ 18 years with any tumor type were eligible, irrespective of disease stage or treatment status, provided they were Dutch‐speaking and able to provide written informed consent. All participants provided written informed consent before study participation. Ethical approval was obtained from the Ethics Committee of the University of Antwerp and UZA on 04‐Jul‐2024 (ID: 5969), in accordance with the Declaration of Helsinki and GDPR.

The quantitative component used a structured multiple‐choice questionnaire addressing ctDNA‐based LB follow‐up (Section A: recall and experiences, Section B: knowledge, and Section C: attitude and preferences), developed from clinically relevant themes informed by practice and literature. Questionnaires were administered in Dutch between 05‐Jul‐2024 and 22‐May‐2025. No formal *a priori* sample size was defined, as the study was exploratory in nature and no single primary hypothesis or effect size was prespecified. To minimize selection bias, patients were randomly approached in waiting rooms and completed paper questionnaires privately on the same day. Questionnaires were retained for the final quantitative analysis only when ≥ 80% of questions in all three sections had been completed. Data were pseudonymized, entered into REDCap®, and supplemented with demographic and clinicopathological data from patient records.

The qualitative component comprised two semi‐structured focus groups: one with expert stakeholders (*n* = 6; clinical geneticist, two medical oncologists, preclinical expert, surgeon, and study coordinator) and one with patient stakeholders (*n* = 4; three female patients respectively with an advanced‐stage gynecological cancer (P3), an early‐stage gynecological cancer (P4), and an early‐stage gastrointestinal (GI) cancer (P2), and one male patient with an advanced‐stage neuroendocrine neoplasm (NEN) (P1)). Focus groups were conducted by three study team members using an interview guide drafted from preliminary questionnaire findings. Sessions were audio‐recorded, transcribed verbatim, translated into English, and quality‐checked.

### 2.2 Statistical analysis

Quantitative data were analyzed descriptively. Categorical variables were reported as counts and percentages. Age at inclusion was summarized as median and range, and was categorized for regression analyses as < 50, 50–70, and > 70 years. Likert‐scale responses were dichotomized as ‘disagree’ (disagree/strongly disagree) and ‘agree’ (neutral/agree/strongly agree). Logistic regression models were used to evaluate associations between patient characteristics and predefined binary questionnaire responses. The following patient characteristics were evaluated as candidate explanatory variables: age group (reference category (ref.): < 50 years), gender (ref.: female), education level (ref.: primary/secondary education), tumor cluster (ref.: gastrointestinal tumors), disease stage (ref.: stage I‐III), and treatment type at inclusion (ref.: chemotherapy). The latter was evaluated as non‐chemotherapy‐based treatment versus chemotherapy treatment. Prior confirmed LB exposure and time since diagnosis were additionally explored in relation to attitude and preference outcomes. Associations were first explored using univariable logistic regression. Given the exploratory nature of the analyses and the limited number of observations in several outcome and predictor categories, a parsimonious, purposeful modeling strategy was applied to avoid overparameterization and unstable estimates. Variables with *P* < 0.10 in univariable analysis or deemed clinically relevant *a priori* were included for multivariable logistic regression models. Model selection was guided by the Akaike Information Criterion, and gender was retained as a covariate in all models. Results were reported as odds ratios (OR) from univariable models and adjusted OR (aOR) from multivariable models, with 95% confidence intervals (CI). ORs and aORs > 1 indicate higher odds of the specified outcome compared with the reference category. Statistical analyses were performed in R (version 4.4.2), with statistical significance set at *P* < 0.05. Focus group transcripts were analyzed using deductive thematic analysis following Braun and Clarke's six‐phase framework [23]. Coding and thematic analysis were performed independently by two researchers using NVivo® (version 14.24.3) and validated by a third, with triangulation across data sources, investigators, and perspectives.

## 3 Results

### 3.1 Patient characteristics and questionnaire overview

A total of 205 patients provided written informed consent. Data from 195 patients with a median age at inclusion of 65 years (range 26–84), who completed ≥ 80% of items in all three sections were retained for analysis. Demographic and clinical characteristics are summarized in Table 1, key themes and subthemes from expert and patient focus groups with illustrative quotes in Table 2, and full questionnaire responses in Table S1.

**Table 1:** Demographics and clinical characteristics. ed., education; GI, gastrointestinal; GYN, gynecological; LB, liquid biopsy; NEN, neuroendocrine neoplasm; PRRT, peptide receptor radionuclide therapy; SSA, somatostatin analog.

Clinical characteristics	Overall (*n* = 195)	GI (*n* = 72)	GYN (*n* = 61)	NEN (*n* = 40)	Other^a^ (*n* = 22)
Age cluster (at inclusion)					
< 50	39 (20%)	11 (15%)	19 (31%)	5 (12%)	4 (18%)
50–70	105 (54%)	40 (56%)	33 (54%)	21 (53%)	11 (50%)
> 70	51 (26%)	21 (29%)	9 (15%)	14 (35%)	7 (32%)
Gender					
Female	123 (63%)	33 (46%)	61 (100%)	20 (50%)	9 (41%)
Male	72 (37%)	39 (54%)	0 (0%)	20 (50%)	13 (59%)
Ethnicity					
European	182 (93%)	66 (92%)	55 (90%)	39 (98%)	22 (100%)
Middle Eastern	5 (3%)	1 (1%)	3 (5%)	1 (2%)	0 (0%)
North African	7 (4%)	4 (6%)	3 (5%)	0 (0%)	0 (0%)
Asian	1 (1%)	1 (1%)	0 (0%)	0 (0%)	0 (0%)
Education level					
Primary and secondary ed.	92 (47%)	37 (51%)	26 (43%)	16 (40%)	13 (60%)
Higher ed. undergraduate	74 (38%)	23 (32%)	29 (48%)	14 (35%)	8 (36%)
Higher ed. postgraduate	29 (15%)	12 (17%)	6 (10%)	10 (25%)	1 (5%)
Disease stage					
Stage I–III	74 (38%)	24 (33%)	34 (56%)	7 (18%)	9 (41%)
Stage IV	121 (62%)	48 (67%)	27 (44%)	33 (83%)	13 (59%)
Participation LB‐based study					
Yes	65 (33%)	24 (33%)	2 (3%)	36 (90%)	3 (14%)
No	130 (67%)	48 (67%)	59 (97%)	4 (10%)	19 (86%)
Treatment (at inclusion)					
Chemotherapy	98 (50%)	49 (68%)	36 (59%)	4 (10%)	9 (41%)
Chemo‐ and immunotherapy	28 (14%)	11 (15%)	8 (13%)	4 (10%)	5 (23%)
Immunotherapy	32 (16%)	6 (8%)	17 (28%)	1 (3%)	8 (36%)
PRRT	4 (2%)	0 (0%)	0 (0%)	4 (10%)	0 (0%)
SSA	12 (6%)	0 (0%)	0 (0%)	12 (30%)	0 (0%)
Targeted therapy	8 (4%)	0 (0%)	0 (0%)	8 (20%)	0 (0%)
Watch and wait	13 (7%)	6 (8%)	0 (0%)	7 (18%)	0 (0%)

a Other tumor types included lung, prostate, and head and neck tumors.

**Table 2:** Overview of themes and subthemes derived from focus groups with illustrative verbatim quotes. Quotes may be shortened for clarity; ellipses (…) indicate omitted text without altering meaning. ctDNA, circulating tumor DNA; LB, liquid biopsy; PPV, positive predictive value; QoL, quality of life.

**Focus group 1 (experts)**		
Theme E1: Cognitive and contextual factors limit recall of LB		
E1.1	Low recall LB is driven by complexity, stress, and context	*“Secondly, patients also don't always remember what happened during a consultation (…), they forget entire parts of it.”* (medical oncologist 1)
E1.2	Simplifying explanations and reinforcing purpose may improve retention	*“They, they will only remember if you make them remember*.” (clinical geneticist)
E1.3	Terminological ambiguity can lead to unintended confusion when explaining LB	“*(…) this term was actually chosen to avoid using the word ‘biopsy’. But yes, it actually creates even more confusion by calling it that.”* (surgeon)
Theme E2: Conceptual complexity constrains understanding of ctDNA and LB		
E2.1	Limited baseline knowledge of ctDNA is expected	*“So I'm not surprised that the number is low.”* (study coordinator)
E2.2	Simplification reduces complexity but does not ensure full understanding of complex scientific concepts	*“And I can't imagine if you're, if you're not using the terms circulating tumor DNA or ctDNA a lot or most of the patients are not going to connect, are not going to connect the dots between what you explained and that specific term.”* (medical oncologist 2)
E2.3	Cognitive overload constrains recall, whereas repetition enhances retention	*“Some of it goes in, some of it goes out, and what goes out are the things that aren't continuously important in their treatment.”* (study coordinator) *“If samples had to be taken continuously (…), but I would expect that they might remember more, because they're continuously reminded of it.”* (study coordinator)
E2.4	Inherent complexity of ctDNA and LB concepts is highlighted by professionals' own learning curves	*“And liquid biopsy, I myself didn't understand that term for a long time.”* (medical oncologist)
E2.5	Communication should be tailored to patients, prioritizing clarity over precision	*“We try to fit our explanation with the patient in front of us.”* (clinical geneticist)
Theme E3: Acceptance of ctDNA‐based LB reflects emotion and context rather than informed preference		
E3.1	Patients trust physicians' advice rather than actively seeking additional information	*“And sometimes some people ask a lot more information. But most of the patients are just thinking, OK, I trust the physician. For me it's fine, just do it.”* (study coordinator)
E3.2	Acceptance of ctDNA‐based LB often reflects anxiety‐driven reassurance	*“(…), what you also test for is anxiety (…).”* (clinical geneticist)
E3.3	Limited contextual understanding constrains informed decision‐making	*“Yeah, but you never talked about sensitivity, specificity, PPV or something like that. So, what do the patients have to answer? They have to answer about acceptance and that's it. They don't have more background.”* (clinical geneticist)
Theme E4: Clinician confidence toward LB shapes patient attitudes		
E4.1	Perceived patient preference is influenced by clinician endorsement, particularly where clinical promise is anticipated	*“In my opinion the patients trust their treating physicians. If you think this this extra blood sample will be an added value or a better evaluation tool, for example, then they will incline to follow it.”* (study coordinator) *“And I would, I would a little bit push on my patients this statement. I would give them information that could push the answer. They would prefer. You would prefer ctDNA. You have to prefer ctDNA above imaging because we have a lot of trials now that say it's prognostic and it's very prognostic.”* (medical oncologist 1)
**Focus group 2 (patients)**		
Theme 1: Recall of LB procedure Is context‐dependent		
P1.1	Recall triggered by cues, not terminology	*“And when I had blood drawn upstairs, I just saw that there were different tubes, and then I asked, ‘Is that for the study?’ and they confirmed it.”* (P4)
P1.2	LB perceived as part of routine care	*“I assume that over the years that extra blood sampling has indeed happened.”* (P3)
P1.3	Recall shaped by disease, treatment, and personal factors	“*I think age. I'm 78, so I don't remember as well as I used to. If they tell me something, I think ‘yes, okay’, but it could well be that a week later I don't remember it, or only half remember.”* (P2) “*It also depends on your level of stress. When you're in active treatment and they propose something, you remember certain things, but a lot doesn't sink in.”* (P4)
P1.4	Support from others can strengthen recall	*“My husband was always with me, so if I missed something, there was always a second person listening. Sometimes he'd say, ‘They said this and that’, and I had forgotten because it hadn't really registered.”* (P3)
P1.5	Familiarity with the (bio)medical field increases research awareness, but does not affect willingness to participate	*“I also had great support from my wife, she's a retired nurse who knows her stuff and knows what to listen for.”* (P1)
Theme 2: Information needs are purpose‐driven rather than knowledge‐driven		
P2.1	Knowing the purpose is good enough, scientific detail is optional	*“Yes, you participate in a study because you think, ‘I can contribute’, (…)”* (P2)
P2.2	More information does not necessarily improve understanding	*“I think that even if I had had more information, I wouldn't have understood it better.’”* (P2)
P2.3	Need for treatment related explanation higher than for research	*“Now, with those studies (referring to LB in research setting) it mattered less for me, but with my treatment I was a bit more bothered by that because I want to know the “why” behind things.”* (P4)
Theme 3: Preferences reflect trust in established diagnostic tools and clinical judgment of healthcare professionals		
P3.1	Preference for established diagnostic modalities	*“Suppose you relapse, it seems to me more black‐and‐white on a scan; you can see it. It's probably also largely the unknown and the new versus something established that's been used for years.”* (P4)
P3.2	LB preferred for lower burden, but scans feel more reliable	*“A blood draw is invasive for a moment, but a scan can also be invasive.”* (P1) *“I assume that if they see something in the blood, the location isn't known? So then a scan is still needed, right?”* (P4)
P3.3	In uncertainty, most decisions default to professional clinical judgment	*“I would always follow the doctor: if they say it's better to do this, then we do this. If they say it's better to do that, then we do that.”* (P2)
Theme 4: Emotional reassurance and practical feasibility shape acceptance		
P4.1	Frequent LB increases reassurance if results are communicated	*“That's the same as with a scan, they let you know, “It was good,” or “It wasn't good.” I would want that same principle. Because, take a scan: you're nervous for a whole week beforehand knowing it's coming up. If you then don't hear anything, you go crazy.”* (P4)
P4.2	LB acceptable only when it does not negatively impact QoL	“*For me, it's (the frequency of LB testing) fine as it is now, because a person also wants to be able to go on holiday, of course.”* (P1)
P4.3	Local sampling and tele‐communication strongly preferred	*“I think a phone call is fine, but if something's wrong, then I'd come in.”* (P4)

### 3.2 Recall accuracy and patient experience with ctDNA‐based LB procedures

Of all participants, 126 reported having undergone a LB, whereas medical records confirmed only 56, yielding a correct recall accuracy of 44%. Participants with correct recall completed the LB recall accuracy and experience questions. Among them, 64% reported receiving additional information from a HCP, perceived as positive (89%) or neutral (11%). In univariable logistic regression, gender, tumor cluster, stage, and treatment type were associated with correct LB recall, defined as self‐reported LB confirmed by the medical records (Fig. 1A). After multivariable adjustment, only tumor cluster remained independently associated with correct LB recall, with NEN patients being more likely to correctly recall LB than participants with GI tumors (aOR 8.04, 95% CI 2.66–24.30, *P* < 0.001, Fig. S1A).

**Fig. 1:**
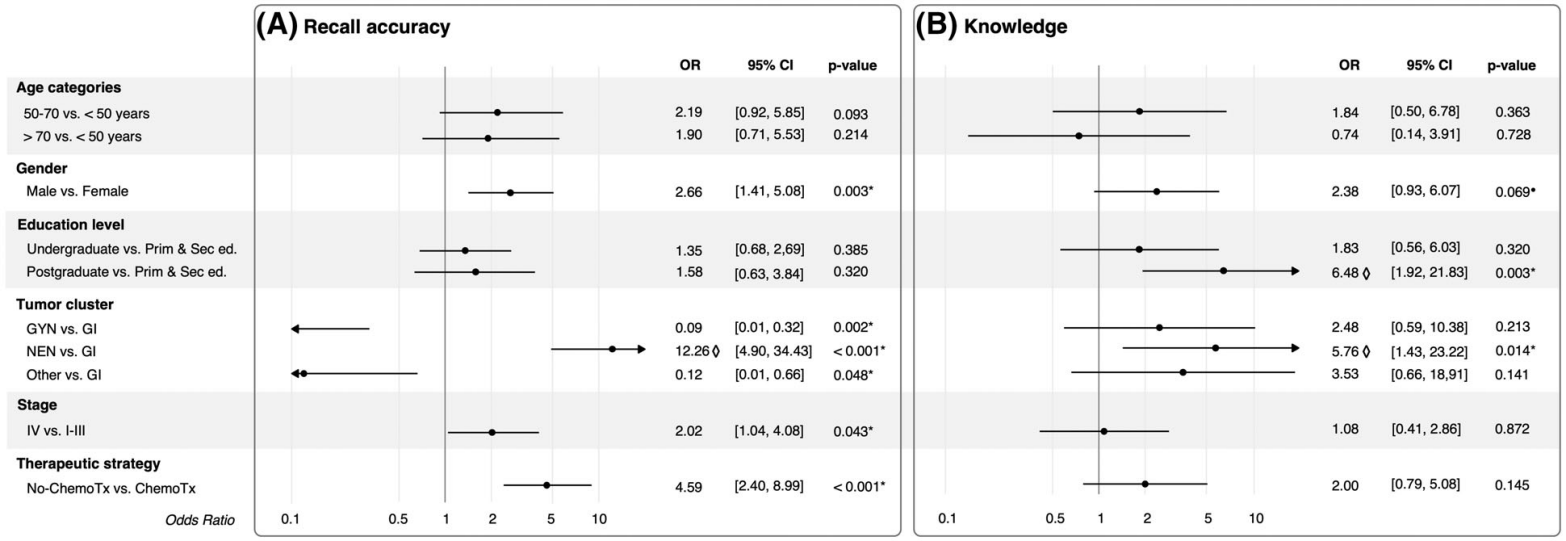
Forest plots illustrating results of univariable analyses showing odds ratios (ORs), corresponding 95% confidence intervals (CIs), and *P*‐values for factors associated with (A) recall accuracy and (B) ctDNA knowledge. Estimates are displayed on a logarithmic scale to allow for consistent comparison across variables. Confidence intervals extending beyond the plotted range are indicated by arrows. Statistically significant associations are denoted with an asterisk (*P < 0.05), suggestive but not statistically significant associations by a dot (●), and variables retained in the multivariable models by a lozenge (◊). ctDNA, circulating tumor DNA; GI, gastrointestinal; GYN, gynecological; NEN, neuroendocrine neoplasm.

Experts attributed low recall to cognitive and contextual factors (Subtheme E1.1 and E1.3; Table S2A): complex consent forms, dense medical explanations, technical terminology, and consultation‐related stress. They recommended simplifying explanations, emphasizing purpose over technical details, and reinforcing verbal information with written materials (Subtheme E1.2), noting that “they will only remember if you make them remember” (clinical geneticist). They also noted that patients rarely request additional information and rely on physician judgment (Subtheme E3.1) and that contextual and cultural factors shaped information‐seeking behavior (Subtheme E3.2), prompting clinicians to proactively verify understanding (Subtheme E2.3). Patients offered a complementary perspective, describing that giving extra blood for LB was indistinguishable from routine care (Subtheme P1.2; Table S2B).

### 3.3 Patients' knowledge of ctDNA and their understanding of its perceived clinical utility

Following a visual explanation, all participants were asked whether they had previously heard of the term ctDNA using a binary (yes/no) question. Only 20 participants (10%) reported prior awareness. In univariable logistic regression, gender, education level, and tumor cluster were associated with prior awareness of the term ctDNA (Fig. 1B). After multivariable adjustment, education level and tumor cluster remained independently associated with prior awareness of the term ctDNA. Participants with postgraduate education were more likely to report prior awareness of ctDNA than those with primary/secondary education (aOR 5.64, 95% CI 1.48–21.52, *P* = 0.011), as were participants with NEN compared with those with GI tumors (aOR 5.16, 95% CI 1.20–22.28, *P* = 0.028). Gynecological (aOR 5.70, 95% CI 0.95–34.32, *P* = 0.057) and other tumor diagnoses (aOR 4.81, 95% CI 0.82–28.28, *P* = 0.082) showed suggestive but not statistically significant associations with greater prior awareness compared with a GI tumor diagnosis (Fig. S1B).

Experts expected limited knowledge of ctDNA and related molecular concepts (Subtheme E2.1). Simplifying explanations was necessary but imperfect (Subtheme E2.2) as information overload and limited perceived relevance constrained retention (Subtheme E2.3). Inherent complexity of these terms was highlighted, as even HCP struggle with them (Subtheme E2.4). Tailoring explanations to patients' comprehension level and prioritizing clarity over precision were emphasized (Subtheme E2.5). Patients echoed these views, as ctDNA was often forgotten despite prior explanation, reflecting cue‐dependent rather than knowledge‐driven recall (Subtheme P1.1). Age, stress, treatment, and external factors impeded retention (Subtheme P1.3), reflecting limited cognitive bandwidth rather than insufficient information. Patients often relied on family members to interpret and remember information (Subtheme P1.4). Familiarity with biomedical concepts increased awareness and interest, not determining willingness to participate (Subtheme P1.5). Across discussions, patients emphasized understanding purpose over scientific mechanisms.

### 3.4 Patients' preferences, attitude, and expectations regarding ctDNA‐based LB testing

All participants were asked to evaluate seven statements exploring their preferences (where a comparison was provided), attitudes, and expectations regarding ctDNA‐based LB. For all statements, no statistically significant differences were observed in time since diagnosis between the dichotomized agree and disagree groups; only numerical differences were observed. Prior LB exposure (participants with correct recall vs. those with incorrect recall) was also evaluated and was not significantly associated with any dichotomized outcome in regression analyses.

#### 3.4.1 Preference and attitude toward ctDNA‐based LB versus imaging

In the first scenario, in which LB could detect progressive disease (PD) or recurrence earlier than imaging, 80% were comfortable with ctDNA‐based LB results guiding clinical action (Fig. 2A). In univariable analysis, age, tumor cluster, and treatment type were associated with comfort with ctDNA‐based LB‐guided clinical action in this scenario (Fig. 3A). After multivariable adjustment, only age remained independently associated with comfort with ctDNA‐based LB‐guided clinical action, while age 50–70 years, tumor cluster, and treatment type showed suggestive, but not statistically significant, associations. Participants aged > 70 years were more likely to be comfortable than those < 50 years (aOR 3.37, 95% CI 1.02–11.11, *P* = 0.046), while participants aged 50–70 years showed a suggestive, but not statistically significant, association with greater comfort compared with those < 50 years (aOR 2.27, 95% CI 0.90–5.72, *P* = 0.082). Non‐chemotherapy treatment showed a suggestive, but not statistically significant, association with lesser comfort compared with chemotherapy treatment (aOR 0.41, 95% CI 0.16–1.05, *P* = 0.063). The same was true for the gynecological tumor diagnosis, which showed a suggestive, but not statistically significant, association with lesser comfort compared with a GI tumor diagnosis (aOR 0.32, 95% CI 0.10–1.00, *P* = 0.050; Fig. S1C).

**Fig. 2:**
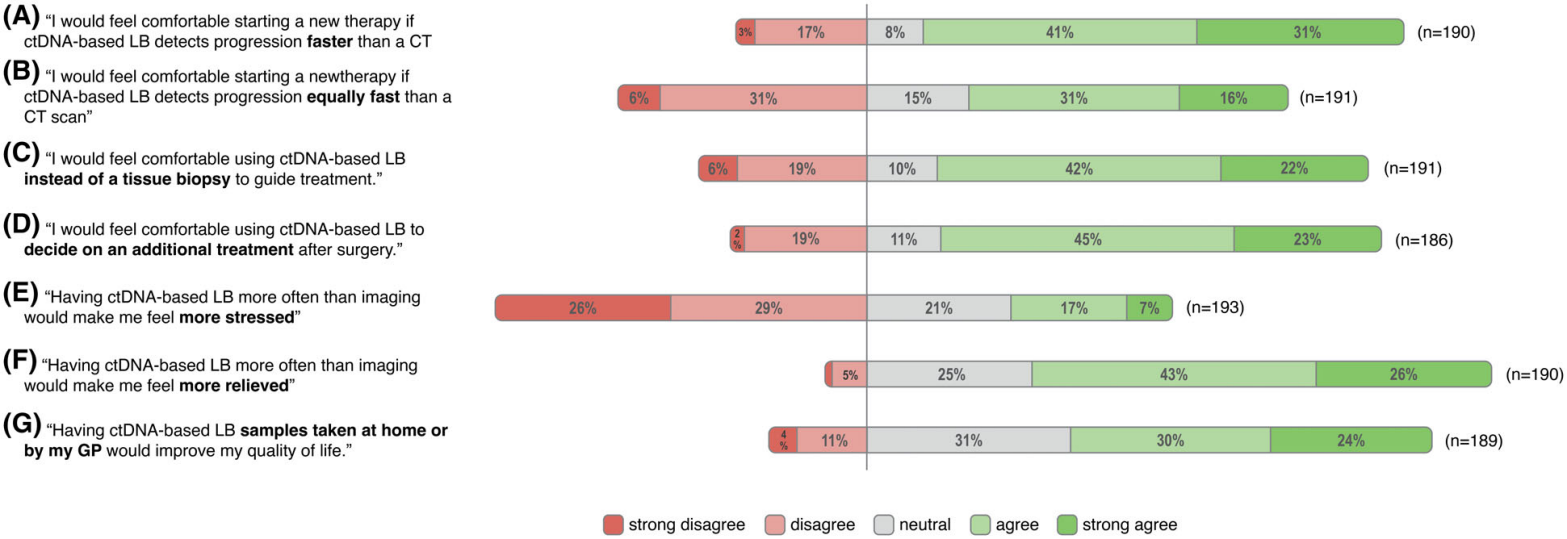
Likert‐style bar plots illustrating participants' responses to seven statements regarding circulating tumor DNA (ctDNA)‐based liquid biopsy testing: (A) clinical action when ctDNA detects progressive disease or recurrence earlier than imaging; (B) clinical action when ctDNA and imaging detect progressive disease or recurrence equally rapidly; (C) use of ctDNA‐based liquid biopsy instead of tissue biopsy; (D) ctDNA‐guided adjuvant treatment in the minimal residual disease setting; (E) perceived stress associated with more frequent testing; (F) perceived reassurance associated with more frequent testing; and (G) perceived quality‐of‐life benefit of home‐ or general practitioner‐based blood sampling. Responses range from “Strongly disagree” to “Strongly agree” and are displayed as the proportion of participants selecting each option. Negative responses are shown to the left of the central axis and neutral or positive responses to the right. Colors indicate response categories, with darker shades representing stronger agreement or disagreement. CT, computed tomography; GP, general practitioner; MRD, minimal residual disease; QoL, quality of life.

**Fig. 3:**
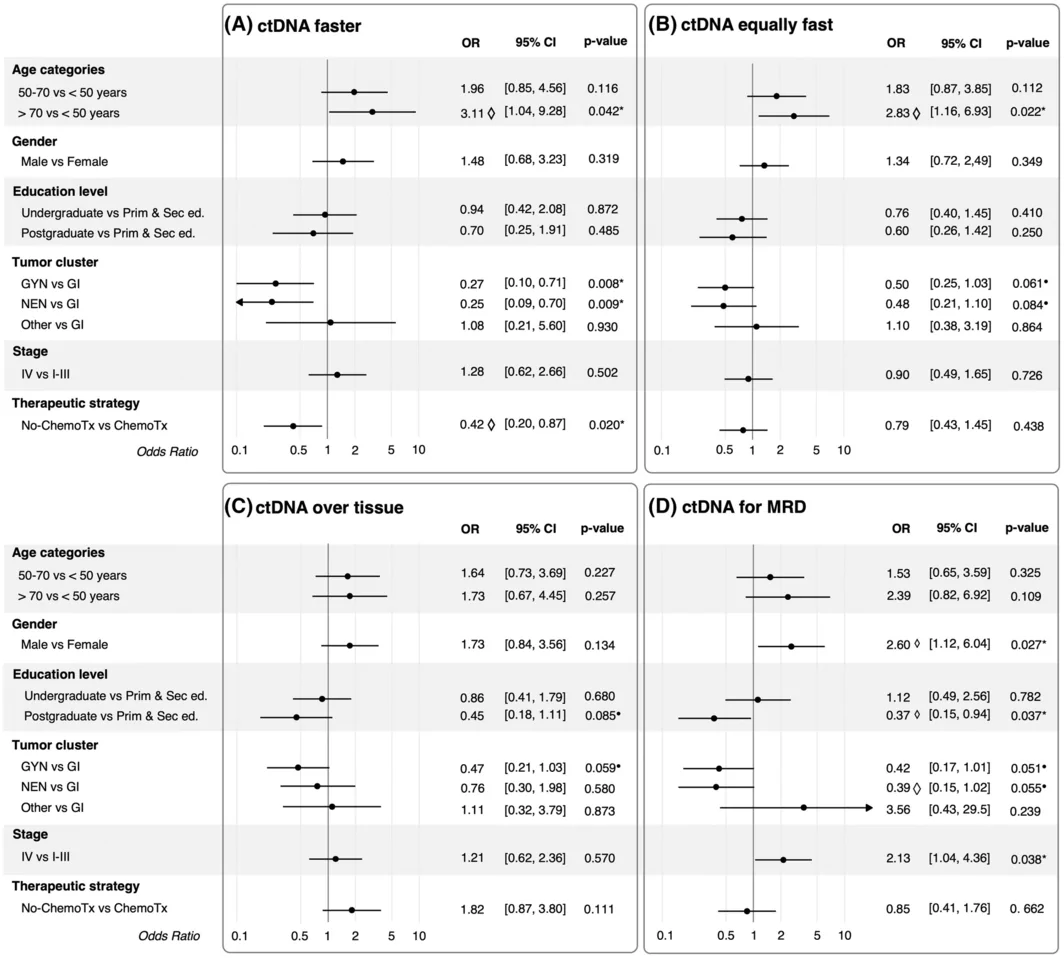
Forest plots of univariable analyses showing odds ratios (ORs) with 95% confidence intervals (CIs) for factors associated with participants' responses regarding the following statements: (A) circulating tumor DNA (ctDNA)‐based liquid biopsy when ctDNA detects progressive disease or recurrence earlier than imaging, (B) ctDNA‐based liquid biopsy when ctDNA and imaging detect progressive disease or recurrence equally rapidly, (C) ctDNA‐based liquid biopsy instead of tissue biopsy, and (D) ctDNA‐guided adjuvant treatment in the minimal residual disease (MRD) setting. Estimates are shown on a logarithmic scale. CIs extending beyond the plotted range are indicated by arrows. Statistically significant associations are denoted by an asterisk (*P < 0.05), suggestive but not statistically significant associations by a dot (●), and variables retained in the multivariable models by a lozenge (◊). GI, gastrointestinal; GYN, gynecological; NEN, neuroendocrine neoplasm.

In the second scenario, in which LB and imaging could detect PD or recurrence equally fast, 62% were comfortable with ctDNA‐based LB‐guided clinical action (Fig. 2B). In univariable analysis, only age was associated with comfort with ctDNA‐based LB‐guided clinical action in this scenario (Fig. 3B). After multivariable adjustment, age remained independently associated with comfort with ctDNA‐based LB‐guided clinical action, while tumor cluster showed a suggestive but not statistically significant association. Participants aged > 70 years were more likely to be comfortable than those aged < 50 years (aOR 2.70, 95% CI 1.06–6.88, *P* = 0.037), while NEN diagnosis showed a suggestive but not statistically significant association with less comfort compared with a GI tumor diagnosis (aOR 0.46, 95% CI 0.20–1.07, *P* = 0.071, Fig. S1D).

Experts emphasized that responses reflect acceptance rather than informed preference (Subtheme E3.1). Earlier detection evokes strong emotions, driving patient endorsement. Acceptance is often anxiety‐driven (Subtheme E3.2), with frequent testing appealing because LB “tests for anxiety” (clinical geneticist). Experts noted that responses would likely shift with more context and if limitations were explicit (Subtheme E3.3).

While patients preferred LB for convenience (Subtheme P3.2), consistent with expert views, this reflected acceptance rather than true preference, as scans remained “much more reliable (P4)” (Subtheme P3.1). Trust in LB increased when it was assumed accurate or faster, despite acknowledged limitations in localization of the anatomical site of the tumoral lesions (Subtheme P3.2). Both modalities were viewed as complementary. Patients deferred decisions to clinicians (Subtheme P3.3): “trust what the doctor says (P4)”, expressing trust‐based acceptance based on perceived accuracy and actionability beyond convenience alone.

#### 3.4.2 Preference and attitude toward ctDNA‐based LB versus tissue

When assessing use of ctDNA‐based LB instead of tissue biopsy (TB) for diagnosis or clinical action, 74% were comfortable, assuming that sufficient clinical evidence would support its use (Fig. 2C). In univariable analysis, education level and tumor cluster showed suggestive, but not statistically significant, associations with comfort with ctDNA‐based LB replacing TB in this scenario (Fig. 3C). After multivariable adjustment, only education level showed a suggestive, but not statistically significant, association. Participants with postgraduate education were less likely to be comfortable with ctDNA‐based LB replacing TB than those with primary/secondary education, although this association was suggestive and not statistically significant (aOR 0.39, 95% CI 0.15–1.02, *P* = 0.054, Fig. S1E).

#### 3.4.3 Preference and attitude toward ctDNA‐based LB in MRD setting

When assessing attitudes toward use of ctDNA to decide on adjuvant treatment in MRD setting, 79% were comfortable (Fig. 2D). In univariable logistic analysis, gender, education level, and disease stage were statistically significantly associated with comfort with ctDNA‐guided adjuvant treatment decisions in the MRD setting (Fig. 3D). After multivariable adjustment, gender, tumor cluster, and education level were independently associated with comfort with ctDNA‐guided adjuvant treatment decisions in the MRD setting. Male participants were more likely to be comfortable than female participants (aOR = 3.12, 95% CI 1.06–9.23, *P* = 0.039), whereas participants with NEN were less likely to be comfortable than those with GI tumors (aOR 0.32, 95% CI 0.11–0.93, *P* = 0.037), and participants with postgraduate education were also less likely to be comfortable than those with primary/secondary education (aOR 0.33, 95% CI 0.12–0.95, *P* = 0.041). Participants with stage IV disease showed a suggestive, but not statistically significant, association with greater comfort compared with those with stage I–III disease (aOR 2.23, 95% CI 0.99–5.03, *P* = 0.054; Fig. S1F).

Experts acknowledged that clinician attitudes and confidence shape patient preference (Subtheme E4.1), with LB more actively promoted in clinically meaningful settings like MRD detection, noting that stronger evidence “would convince [patients] more to use this technique” (medical oncologist 1). Patients reflected this dynamic, deferring adjuvant treatment decisions to clinicians, consistent with Subtheme P3.3.

#### 3.4.4 Stress and relief related to ctDNA‐based LB

When assessing the emotional impact, 55% reported that more frequent testing would not increase stress (Fig. 2E), with no statistically significant demographic associations observed. Conversely, 94% reported that more frequent testing would provide relief (Fig. 2F). In univariable analysis, participants receiving non‐chemotherapy treatment were less likely to report relief than those receiving chemotherapy (OR 0.25, 95% CI 0.07–0.86, *P* = 0.028) (Fig. S2B). Treatment type also showed a suggestive, but not statistically significant, association with perceived stress, with participants receiving non‐chemotherapy treatment being more likely to report increased stress than those receiving chemotherapy (OR 1.71, 95% CI 0.94–3.10, *P* = 0.079) (Fig. S2A). After multivariable adjustment, participants receiving non‐chemotherapy treatment remained less likely to report relief than those receiving chemotherapy (aOR 0.26, 95% CI 0.07–0.89, *P* = 0.032; Fig. S1G). For perceived stress, treatment type remained suggestively, but not statistically significantly, associated with increased stress among participants receiving non‐chemotherapy compared with chemotherapy (aOR 1.71, 95% CI 0.94–3.11, *P* = 0.080; Fig. S1H).

Patients noted that frequent ctDNA testing was described as inherently reassuring, driven more by anxiety reduction through regular confirmation than clinical benefit (Subtheme P4.1), echoing Subtheme E3.2 from the expert focus group.

#### 3.4.5 Practical preferences and communication related to ctDNA

Finally, participants were asked about the practicality of ctDNA‐based LB testing in routine care. The majority (85%) believed that home‐ or GP‐based LB sampling would improve their quality of life (QoL) (Fig. 2G), with no significant demographic associations observed (Fig. S3).

Patient focus group reinforced that frequent LB sampling was acceptable if QoL was preserved through flexible scheduling (Subtheme P4.2). Patients strongly preferred ambulant sampling (e.g. home) with telephone‐based communication of results, reserving in‐person visits for clinical follow‐up (Subtheme P4.3).

Regarding requirements for reliable ctDNA‐based LB testing, participants most frequently emphasized accuracy (83%) and speed of detection (43%). Nearly all (92%) wished to be informed of the results, with another 4% preferring notification of favorable results only; 94% preferred receiving information directly via an HCP rather than through printed or digital materials.

## 4 Discussion

This mixed‐methods study provides new insights into patients' perspectives on ctDNA‐based LB in oncological care. Only 44% of patients correctly recalled prior ctDNA‐based LB exposure and 10% had heard of ctDNA, consistent with prior reports of limited patient understanding of LB [21] and underlying genomic testing‐related concepts in cancer patients [24, 25]. This low awareness may partly reflect differences in terminology used in clinical practice, but still points to limited understanding of genomic concepts. Higher education was associated with better ctDNA knowledge, aligning with prior evidence [24]. Patients with NEN had better recall and knowledge, possibly due to lower distress, longer follow‐up facilitating retention, and higher LB exposure within research settings. The discrepancy between reported and correctly recalled LB exposure may be explained by ambiguity in the terminology, with patients potentially considering routine blood draws rather than ctDNA‐based testing. Standardized communication, such as structured explanations embedded in clinical workflows or patient information processes, may support the future integration of LB into standard‐of‐care clinical practice.

Qualitatively, experts highlighted cognitive and contextual barriers to recall, while patients described LB as indistinguishable from routine blood draws, reflecting cue‐dependent rather than knowledge‐driven recall. This reflects the Belgian context, where LB remains research‐only and therefore less salient. Information overload and low perceived relevance further impeded retention, with difficulties attributed to age, treatment burden, and emotional stress. This is consistent with established evidence on chemotherapy and age‐related cognitive impairment [26, 27], and broader cancer‐ and stress‐associated cognitive dysfunction [28]. As LB enters standard‐of‐care, clearer distinction may improve recognition; however, our findings challenge the assumption whether this persistent knowledge gap must, or can, be eliminated, given patients' emphasis on purpose, its impact on care, and trust in clinicians, rather than technical understanding. This aligns with evidence showing that understanding test purpose and rationale enhances acceptance and reduces uncertainty [29]. A pragmatic, purpose‐driven, non‐technical communication approach, supported by printed or digital materials, may foster meaningful understanding while accommodating individual needs.

Attitudes toward ctDNA‐based LB were favorable across clinical contexts. Acceptance was context‐dependent, highest when LB was perceived as faster than or comparable to imaging, and remained high relative to TB. Accuracy and speed were consistently emphasized as key requirements for reliability, extending prior findings [21]. Older participants were more comfortable with LB‐guided care, possibly reflecting a preference for less burdensome procedures. Patients with NEN were less accepting than those with GI tumors, despite better recall, knowledge, and LB exposure in NEN patients, suggesting differing clinical urgency or follow‐up needs. Conversely, participants with postgraduate education were slightly less comfortable with LB replacing TB, possibly reflecting concerns regarding accuracy, indicating that perspectives on ctDNA‐based LB vary by both clinical and individual factors.

Experts interpreted favorable patient responses with caution, suggesting that preferences may shift if limitations and downstream actionability are clearly communicated. They also considered that favorable responses may partly reflect anxiety‐driven acceptance rather than fully informed preference. This interpretation is plausible, as emotional appeal and reassurance may amplify enthusiasm, with prior evidence linking scan‐related anxiety to positive attitudes toward ctDNA surveillance [30]. However, this was only partly supported by the patients. LB was valued for its comfort and ease, yet imaging remained the perceived benchmark, with most favoring a complementary approach. Moreover, their acceptance was not unconditional but depended on perceived accuracy, clinical usefulness, preserved quality of life, and clinician guidance. Thus, while expert and patient perspectives aligned in recognizing reassurance and trust as important drivers, patients' responses challenged a purely anxiety‐based interpretation and highlighted the importance of perceived clinical value. Clinician trust and guidance shaped acceptability, as patients often deferred decisions despite uncertainty, consistent with evidence that physician trust contributes to patient acceptability even without detailed information in early‐stage cancer care [31].

Patients' attitudes were also shaped by emotional and practical considerations as most perceived frequent ctDNA‐based LB as inherently reassuring rather than stress‐inducing, in line with evidence that cancer survivors value frequent follow‐up for emotional security [32] and that LB may reduce anxiety in endometrial cancer follow‐up [30]. Nearly all participants wished to receive their ctDNA results, preferably through direct communication with an HCP. Acceptability of frequent ctDNA‐based LB sampling depended on preserved QoL, with preferences for flexible scheduling, home‐based sampling, and brief communication of routine results. These findings reflect patients' high value on diagnostic certainty and timely information, consistent with prior work showing that full disclosure of disease status enhances sense of control and symptom management [33].

Our mixed‐methods study advances understanding of patients' perspectives on ctDNA‐based LB by showing that limited recall and conceptual understanding can coexist with high purpose‐driven acceptance. By examining multiple clinical scenarios, we demonstrate that acceptance is conditional rather than static, in contrast to prior assessments limited to single hypothetical settings [21]. Inclusion of a substantial subgroup with prior LB experience revealed better recall and knowledge but not consistently higher acceptance, indicating that exposure alone is insufficient without perceived clinical value. Although emerging evidence [34, 35] supports the role of ctDNA‐based LB in oncology, major guidelines remain cautious pending proof of clinical utility, which remains highly tumor type and indication specific. Broader implementation will likely depend on robust actionability, well‐defined use cases, and clinician confidence, with MRD detection representing a potential early, patient‐aligned entry point.

This single‐center study in an academic hospital may limit generalizability. The cohort consisted predominantly of patients of European ethnicity and included only small numbers of several common cancer types, with no breast cancer patients and only a limited number of patients with lung and prostate cancer, which may further restrict the broader applicability of the findings. Additionally, the cohort included a relatively high proportion of patients with NEN, many of whom had prior exposure to LB, introducing potential imbalance and limiting generalizability to other cancer populations. While this introduces imbalance, it also provides valuable insight from patients with real LB experience. The study was conducted among Dutch‐speaking patients in Belgium, where ctDNA‐based testing is only used for research purposes. Patient recall, knowledge, and attitudes may therefore differ in countries where ctDNA‐based testing is already more embedded in standard care, commercially available, or supported by different reimbursement structures. Attitudes toward ctDNA‐based LB also may differ in real‐world decision‐making compared with hypothetical scenarios, and responses may have been influenced by social desirability or unfamiliarity with the concept. Finally, the qualitative component included a limited number of experts and patients, limiting the extent to which focus group findings can be generalized. Nonetheless, the mixed‐methods approach allows triangulation of quantitative and qualitative findings and provides a comprehensive understanding of patient perspectives.

## 5 Conclusion

Despite limited recall and conceptual knowledge, patients generally viewed ctDNA‐based LB favorably, recognized its benefits, and expressed clear expectations regarding accuracy, aligning closely with HCP perspectives. High acceptance of ctDNA‐based LB was primarily driven by perceived purpose, anticipated clinical impact, emotional reassurance, and trust in clinicians rather than technical understanding. Preferences were context‐dependent and shaped by clinician framing and perceived added value, indicating a strong foundation for patient‐aligned implementation of ctDNA‐based LB as evidence of clinical utility continues to evolve. Our findings highlight the importance of pragmatic, purpose‐driven communication and co‐constructed decision‐making in this process.

## Conflict of interest

The authors declare no conflict of interest.

## Author contributions

SC and LM were involved in conceptualization, data curation, formal analysis, investigation, methodology, project administration, visualization, and writing the original draft. CDW and SW were involved in conceptualization, investigation, methodology, and reviewing and editing the manuscript. LAT and HP were involved in reviewing and editing the manuscript. KJ was involved in conceptualization, methodology, supervision, and reviewing and editing the manuscript. TV was involved in conceptualization, methodology, funding acquisition, resources, supervision, and reviewing and editing the manuscript. All authors approved the final version of the manuscript.

## Supporting information


**Table S1.** Questionnaire responses investigating patients' perspectives on liquid biopsy and circulating tumor DNA.
**Table S2.** (A) Themes and subthemes derived from the expert focus group. (B) Themes and subthemes derived from the patient focus group.
**Fig. S1.** Multivariable analyses of factors associated with recall accuracy, ctDNA knowledge, and attitudes toward ctDNA‐based liquid biopsy.
**Fig. S2.** Univariable analyses of factors associated with stress and relief related to more frequent ctDNA‐based testing.
**Fig. S3.** Univariable analysis of factors associated with perceived quality‐of‐life improvement with home‐ or general practitioner‐based ctDNA sampling.

## Data Availability

The translated questions from the questionnaires and the corresponding response frequencies are provided in Table S1. The interview and online survey data that support the findings of this study are available from the corresponding author upon reasonable request.
