# Supplementary material for: Attitudes of cancer patients toward ctDNA‐based liquid biopsy for follow‐up
Source: Mol Oncol. 2026 Oct 21:10.1002/1878-0261.70352. Online ahead of print. doi: 10.1002/1878-0261.70352 (PMC13685910; doi:10.1002/1878-0261.70352)
Supplement: Supplementary file 1 [file MOL2-9999-0-s001.docx]

| **Supplementary table 1. Questionnaire responses investigating patients’ perspectives on liquid biopsy and circulating tumor DNA.** | | | |
| --- | --- | --- | --- |
| **Question** | **Respondents (n)** | **Response options** | **Frequency (%)** |
| *Part 1: Questions related to recall and experience related to LB procedure were asked* | | | |
| 1. Has an additional blood sample even been taken at any point during the follow-up of your cancer? | 195 | Yes | 64.6% |
|  |  | No | 35.4% |
| 2. When was the last additional blood sample taken during your cancer follow-up? *(conditional on a ‘yes’ response to question 1)* | 126 | 0-3 months | 74.6% |
|  |  | 3-6 months | 9.5% |
|  |  | 6-12 months | 1.6% |
|  |  | > 12 months | 6.3% |
|  |  | I don’t know | 7.9% |
| 3. Did you receive any information at the time these additional (blood) samples were taken? *(conditional on a ‘yes’ response to question 1)* | 126 | Yes | 71.4% |
|  |  | No | 28.6% |
| 4. If yes, who provided this information? *(conditional on a ‘yes’ response to question 3)* | 90 | Physician | 65.6% |
|  |  | Nurse | 18.9% |
|  |  | Study coordinator | 17.8% |
|  |  | Oncocoach | 8.9% |
| 5. How would you rate your experience with the information you received? *(conditional on a ‘yes’ response to question 1)* | 86 | very poor | 1.2% |
|  |  | poor | 2.3% |
|  |  | neutral | 10.5% |
|  |  | good | 55.8% |
|  |  | very good | 30.2% |
| 6. Did you know the purpose of these additional (blood samples)? If yes, for what purpose? *(multiple responses possible, conditional on a ‘yes’ response to question 1)* | 123 | yes – research | 56.1% |
|  |  | yes – treatment | 30.1% |
|  |  | yes – follow-up | 21.9% |
|  |  | yes – other | 8.1% |
|  |  | no | 8.9% |
| 7. How were these samples referred to? *(multiple responses possible, conditional on a ‘yes’ response to question 1)* | 123 | Blood sample(s) (Dutch) | 66.7% |
|  |  | Fluid biopsy (Dutch) | 4.1% |
|  |  | Liquid biopsy | 2.4% |
|  |  | Study sample | 9.8% |
|  |  | Other^1^ / I don’t know | 24.4% |
| 8. How would you rate your overall experience with the way these additional samples were taken? *(conditional on a ‘yes’ response to question 1)* | 124 | very poor | 0.00% |
|  |  | poor | 0.81% |
|  |  | neutral | 13.7% |
|  |  | good | 50.0% |
|  |  | very good | 35.5% |
| *Part 2: Questions related to recall ctDNA and preferences for ctDNA-based LB were asked (concept of ctDNA and ctDNA-based liquid biopsies was explained through patient-friendly illustration)* | | | |
| 9. In addition to blood, where do you think ctDNA (biomarker) could be detected? *(multiple responses possible)* | 195 | Urine | 27.2% |
|  |  | Cerebrospinal fluid | 20.0% |
|  |  | Stool | 21.5% |
|  |  | Saliva | 17.9% |
|  |  | Other | 9.7% |
|  |  | I don’t know | 49.7% |
| 10. Had you ever heard of the term ‘ctDNA’ (= circulating tumor DNA) before this survey? | 191 | yes | 10.5% |
|  |  | no | 89.5% |
| 11. After receiving background information, for which purpose do you think ctDNA could be used in oncology? *(multiple responses possible)* | 194 | For diagnosis | 55.2% |
|  |  | For treatment selection | 64.4% |
|  |  | For treatment monitoring | 56.2% |
|  |  | For deciding on adjuvant treatment after surgery | 36.6% |
|  |  | Other | 2.1% |
|  |  | I don’t know | 21.1% |
| 12. Under which conditions would ctDNA-based testing be considered reliable for cancer follow-up in your opinion? *(multiple responses possible)* | 189 | Must be accurate | 82.5% |
|  |  | Must detect disease progression faster | 43.4% |
|  |  | Must cause less discomfort | 18.0% |
|  |  | Must be performed more frequently | 19.6% |
|  |  | Must be possible to collect samples at home/by GP | 22.2% |
| *Part 3: Statements were presented, exploring preferences related to ctDNA-based LB in various hypothetical clinical scenario’s* | | | |
| 13. If ctDNA testing could detect cancer recurrence or progression **faster** than standard imaging, would you feel comfortable starting a new therapy based on ctDNA results alone? | 190 | strongly disagree | 2.6% |
|  |  | disagree | 16.8% |
|  |  | neutral | 8.4% |
|  |  | agree | 41.1% |
|  |  | strongly agree | 31.1% |
| 14. If ctDNA testing were **equally fast** as standard imaging in detecting disease progression, would you feel comfortable starting a new therapy based on ctDNA results alone? | 191 | strongly disagree | 6.3% |
|  |  | disagree | 30.9% |
|  |  | neutral | 15.2% |
|  |  | agree | 31.4% |
|  |  | strongly agree | 16.2% |
| 15. If ctDNA testing were used **instead of a tissue biopsy** to guide treatment decisions, would you feel comfortable with this approach? | 191 | strongly disagree | 5.8% |
|  |  | disagree | 19.4% |
|  |  | neutral | 10.5% |
|  |  | agree | 42.4% |
|  |  | strongly agree | 22.0% |
| 16. If ctDNA testing were used **after surgery** to decide whether you need additional treatment, would you feel comfortable or satisfied with this approach? | 186 | strongly disagree | 2.2% |
|  |  | disagree | 18.8% |
|  |  | neutral | 11.3% |
|  |  | agree | 45.2% |
|  |  | strongly agree | 22.6% |
| 17. Would you like to be informed of your ctDNA test results? | 189 | yes | 92.1% |
|  |  | yes, but only if reassuring | 3.7% |
|  |  | no | 1.1% |
|  |  | I don’t know | 3.2% |
| 18. If ctDNA were measured more frequently (e.g., monthly) instead of at longer intervals between CT/MRI scans (typically every 2-3 months): Would this cause you **more stress**? | 193 | strongly disagree | 25.9% |
|  |  | disagree | 29.0% |
|  |  | neutral | 21.2% |
|  |  | agree | 17.1% |
|  |  | strongly agree | 6.7% |
| 19. If ctDNA were measured more frequently (e.g., monthly) instead of at longer intervals between CT/MRI scans (typically every 2-3 months): Would this cause you **more reassurance**? | 190 | strongly disagree | 1.1% |
|  |  | disagree | 5.3% |
|  |  | neutral | 24.7% |
|  |  | agree | 42.6% |
|  |  | strongly agree | 26.3% |
| 20. If ctDNA could be measured at home or by your general practitioner, avoiding hospital visits, would this improve your quality of life? | 189 | strongly disagree | 4.2% |
|  |  | disagree | 10.6% |
|  |  | neutral | 30.7% |
|  |  | agree | 30.2% |
|  |  | strongly agree | 24.3% |
| 21. How would you prefer to receive information about ctDNA testing and its use in cancer follow-up? *(multiple responses possible)* | 192 | Direct communication with healthcare professional | 93.8% |
|  |  | Information brochure | 18.8% |
|  |  | Video | 5.7% |
|  |  | Other | 2.1% |
| **Footnotes:** ^1^Terminology referred to in 'others' section of question 7 (genetics, genetic test abnormality, biobank, genetic testing, infection, whole genome sequencing, high fever, check-up, cancer); ^2^Terminology referred to in 'others' section of question 9 (bone marrow, skin, mucosa, all tissues, biopsies, organs (e.g. liver, kidney, lymph nodes), tumor, tissue, cancer cell biopsy, blood, sperm); ^3^Terminology referred to in 'others' section of question 11 (at relapse, genetics); | | | |
| **Abbreviations:** CT, computed tomography; ctDNA, circulating tumor DNA; GP, general practitioner; LB, liquid biopsy; MRI, magnetic resonance imaging. | | | |

| **Supplementary Table 2A.** Themes and subthemes derived from the expert focus group, with illustrative verbatim quotes. Quotes may be shortened for clarity; ellipses (…) indicate omitted text without altering meaning. Abbreviations: ctDNA, circulating tumor DNA; LB, liquid biopsy; PPV, positive predictive value. | | |
| --- | --- | --- |
| **Theme E1: Cognitive and contextual factors limit recall of LB** | | |
| E1.1 | Low recall LB is driven by complexity, stress, and context | *“Secondly, patients also don’t always remember what happened during a consultation (…), they forget entire parts of it.”* (medical oncologist 1) |
| E1.2 | Simplifying explanations and reinforcing purpose may improve retention | *“They, they will only remember if you make them remember*.” (clinical geneticist) |
| E1.3 | Terminological ambiguity can lead to unintended confusion when explaining LB | “*(…) this term was actually chosen to avoid using the word ‘biopsy’. But yes, it actually creates even more confusion by calling it that.”* (surgeon) |
| **Theme E2: Conceptual complexity constrains understanding of ctDNA and LB** | | |
| E2.1 | Limited baseline knowledge of ctDNA is expected | *“So I’m not surprised that the number is low.”* (study coordinator) |
| E2.2 | Simplification reduces complexity but does not ensure full understanding of complex scientific concepts | *“And I can't imagine if you're, if you're not using the terms circulating tumor DNA or ctDNA a lot or most of the patients are not going to connect, are not going to connect the dots between what you explained and that specific term.”* (medical oncologist 2) |
| E2.3 | Cognitive overload constrains recall, whereas repetition enhances retention | *“Some of it goes in, some of it goes out, and what goes out are the things that aren’t continuously important in their treatment.”* (study coordinator)  *“If samples had to be taken continuously (…), but I would expect that they might remember more, because they’re continuously reminded of it.”* (study coordinator) |
| E2.4 | Inherent complexity of ctDNA and LB concepts is highlighted by professionals’ own learning curves | *“And liquid biopsy, I myself didn’t understand that term for a long time.”* (medical oncologist) |
| E2.5 | Communication should be tailored to patients, prioritizing clarity over precision | *“We try to fit our explanation with the patient in front of us.”* (clinical geneticist) |
| **Theme E3: Acceptance of ctDNA-based LB reflects emotion and context rather than informed preference** | | |
| E3.1 | Patients trust physicians’ advice rather than actively seeking additional information | *“And sometimes some people ask a lot more information. But most of the patients are just thinking, OK, I trust the physician. For me it's fine, just do it.”* (study coordinator) |
| E3.2 | Acceptance of ctDNA-based LB often reflects anxiety-driven reassurance | *“(…), what you also test for is anxiety (…).”* (clinical geneticist) |
| E3.3 | Limited contextual understanding constrains informed decision-making | *“Yeah, but you never talked about sensitivity, specificity, PPV or something like that. So, what do the patients have to answer? They have to answer about acceptance and that's it. They don't have more background.”* (clinical geneticist) |
| **Theme E4: Clinician confidence toward LB shapes patient attitudes** | | |
| E4.1 | Perceived patient preference is influenced by clinician endorsement, particularly where clinical promise is anticipated | *“In my opinion the patients trust their treating physicians. If you think this this extra blood sample will be an added value or a better evaluation tool, for example, then they will incline to follow it.”* (study coordinator)  *“And I would, I would a little bit push on my patients this statement. I would give them information that could push the answer. They would prefer. You would prefer ctDNA. You have to prefer ctDNA above imaging because we have a lot of trials now that say it's prognostic and it's very prognostic.”* (medical oncologist 1) |

| **Supplementary Table 2B.** Themes and subthemes derived from the patient focus group, with illustrative verbatim quotes. Quotes may be shortened for clarity; ellipses (…) indicate omitted text without altering meaning. Abbreviations: LB, liquid biopsy; QoL, quality of life. | | |
| --- | --- | --- |
| **Theme 1: Recall of LB procedure Is context-dependent** | | |
| P1.1 | Recall triggered by cues, not terminology | *“And when I had blood drawn upstairs, I just saw that there were different tubes, and then I asked, ‘Is that for the study?’ and they confirmed it.”* (P4) |
| P1.2 | LB perceived as part of routine care | *“I assume that over the years that extra blood sampling has indeed happened.”* (P3) |
| P1.3 | Recall shaped by disease, treatment, and personal factors | “*I think age. I’m 78, so I don’t remember as well as I used to. If they tell me something, I think ‘yes, okay’, but it could well be that a week later I don’t remember it, or only half remember.”* (P2)  “*It also depends on your level of stress. When you’re in active treatment and they propose something, you remember certain things, but a lot doesn’t sink in.”* (P4) |
| P1.4 | Support from others can strengthen recall | *“My husband was always with me, so if I missed something, there was always a second person listening. Sometimes he’d say, ‘They said this and that’, and I had forgotten because it hadn’t really registered.”* (P3) |
| P1.5 | Familiarity with the (bio)medical field increases research awareness, but does not affect willingness to participate | *“I also had great support from my wife, she’s a retired nurse who knows her stuff and knows what to listen for.”* (P1) |
| **Theme 2: Information needs are purpose-driven rather than knowledge-driven** | | |
| P2.1 | Knowing the purpose is good enough, scientific detail is optional | *“Yes, you participate in a study because you think, ‘I can contribute’, (…)”* (P2) |
| P2.2 | More information does not necessarily improve understanding | *“I think that even if I had had more information, I wouldn’t have understood it better.’”* (P2) |
| P2.3 | Need for treatment related explanation higher than for research | *“Now, with those studies (referring to LB in research setting) it mattered less for me, but with my treatment I was a bit more bothered by that because I want to know the “why” behind things.”* (P4) |
| **Theme 3: Preferences reflect trust in established diagnostic tools and clinical judgement of healthcare professionals** | | |
| P3.1 | Preference for established diagnostic modalities | *“Suppose you relapse, it seems to me more black-and-white on a scan; you can see it. It’s probably also largely the unknown and the new versus something established that’s been used for years.”* (P4) |
| P3.2 | LB preferred for lower burden, but scans feel more reliable | *“A blood draw is invasive for a moment, but a scan can also be invasive.”* (P1)  *“I assume that if they see something in the blood, the location isn’t known? So then a scan is still needed, right?”* (P4) |
| P3.3 | In uncertainty, most decisions default to professional clinical judgement | *“I would always follow the doctor: if they say it’s better to do this, then we do this. If they say it’s better to do that, then we do that.”* (P2) |
| **Theme 4: Emotional reassurance and practical feasibility shape acceptance** | | |
| P4.1 | Frequent LB increases reassurance if results are communicated | *“That’s the same as with a scan, they let you know, “It was good,” or “It wasn’t good.” I would want that same principle. Because, take a scan: you’re nervous for a whole week beforehand knowing it’s coming up. If you then don’t hear anything, you go crazy.”* (P4) |
| P4.2 | LB acceptable only when it does not negatively impact QoL | “*For me, it’s (the frequency of LB testing) fine as it is now, because a person also wants to be able to go on holiday, of course.”* (P1) |
| P4.3 | Local sampling and tele-communication strongly preferred | *“I think a phone call is fine, but if something’s wrong, then I’d come in.”* (P4) |

**Supplementary Figure 1.** **Forest plots illustrating results of multivariable analyses showing adjusted odds ratios (aORs), corresponding 95% confidence intervals (CIs), and p values for factors associated with (A) recall accuracy, (B) circulating tumor DNA (ctDNA) knowledge, and responses regarding (C) ctDNA-based liquid biopsy when ctDNA detects disease progression or recurrence earlier than imaging, (D) ctDNA-based liquid biopsy when ctDNA and imaging detect disease progression or recurrence equally rapidly, (E) ctDNA-based liquid biopsy instead of tissue biopsy, (F) ctDNA-guided adjuvant treatment in the minimal residual disease (MRD) setting, (G) relief, and (H) stress as a consequence of ctDNA-based testing.** Estimates are displayed on a logarithmic scale to allow consistent comparison across variables. Confidence intervals extending beyond the plotted range are indicated by arrows. Adjusted estimates are shown for all variables included in each respective multivariable model. Variables displayed reflect those retained according to the prespecified purposeful model-building strategy and therefore may differ between models. Statistically significant associations are denoted by an asterisk (* p < 0.05), and suggestive but not statistically significant associations by a dot (●). GI, gastrointestinal; GYN, gynecological; NEN, neuroendocrine neoplasm.

**Supplementary Figure 2. Forest plots illustrating results of univariable analyses showing odds ratios (ORs), corresponding 95% confidence intervals (CIs), and p values for factors associated with (A) stress and (B) relief as a consequence of ctDNA-based testing.** Estimates are displayed on a logarithmic scale to allow consistent comparison across variables. Confidence intervals extending beyond the plotted range are indicated by arrows. Statistically significant associations are denoted by an asterisk (* p < 0.05), and suggestive but not statistically significant associations by a dot (●). GI, gastrointestinal; GYN, gynecological; NEN, neuroendocrine neoplasm.

**Supplementary Figure 3. Forest plot illustrating results of univariable analyses showing odds ratios (ORs), corresponding 95% confidence intervals (CIs), and p values for factors associated with perceived improvement in quality of life with home- or general practitioner (GP)-based circulating tumor DNA (ctDNA) sampling.** Estimates are displayed on a logarithmic scale to allow consistent comparison across variables. Confidence intervals extending beyond the plotted range are indicated by arrows. Statistically significant associations are denoted by an asterisk (* p < 0.05), and suggestive but not statistically significant associations by a dot (●). GI, gastrointestinal; GYN, gynecological; NEN, neuroendocrine neoplasm.
